# Impairments in reinforcement learning do not explain enhanced habit formation in cocaine use disorder

**DOI:** 10.1007/s00213-019-05330-z

**Published:** 2019-08-01

**Authors:** T. V. Lim, R. N. Cardinal, G. Savulich, P. S. Jones, A. A. Moustafa, T. W. Robbins, K. D. Ersche

**Affiliations:** 1 Departments of Psychiatry, Psychology and Clinical Neurosciences, University of Cambridge, Herchel Smith Building for Brain & Mind Sciences, Cambridge Biomedical Campus, Cambridge, CB2 0SZ UK; 2 Behavioural and Clinical Neurosciences Institute, University of Cambridge, Cambridge, UK; 3 Liaison Psychiatry Service, Cambridgeshire & Peterborough NHS Foundation Trust, Box 190, Cambridge Biomedical Campus, Cambridge, CB2 0QQ UK; 4 School of Social Sciences and Psychology, MARCS Institute for Brain and Behaviour, Western Sydney University, Sydney, NSW Australia

**Keywords:** Goal-directed learning/behaviour, Habit, Computational modelling, Hierarchical Bayesian, Appetitive discrimination learning, Reinforcement sensitivity, Positive feedback, Extinction, Perseveration

## Abstract

**Rationale:**

Drug addiction has been suggested to develop through drug-induced changes in learning and memory processes. Whilst the initiation of drug use is typically goal-directed and hedonically motivated, over time, drug-taking may develop into a stimulus-driven habit, characterised by persistent use of the drug irrespective of the consequences. Converging lines of evidence suggest that stimulant drugs facilitate the transition of goal-directed into habitual drug-taking, but their contribution to goal-directed learning is less clear. Computational modelling may provide an elegant means for elucidating changes during instrumental learning that may explain enhanced habit formation.

**Objectives:**

We used formal reinforcement learning algorithms to deconstruct the process of appetitive instrumental learning and to explore potential associations between goal-directed and habitual actions in patients with cocaine use disorder (CUD).

**Methods:**

We re-analysed appetitive instrumental learning data in 55 healthy control volunteers and 70 CUD patients by applying a reinforcement learning model within a hierarchical Bayesian framework. We used a regression model to determine the influence of learning parameters and variations in brain structure on subsequent habit formation.

**Results:**

Poor instrumental learning performance in CUD patients was largely determined by difficulties with learning from feedback, as reflected by a significantly reduced learning rate. Subsequent formation of habitual response patterns was partly explained by group status and individual variation in reinforcement sensitivity. White matter integrity within goal-directed networks was only associated with performance parameters in controls but not in CUD patients.

**Conclusions:**

Our data indicate that impairments in reinforcement learning are insufficient to account for enhanced habitual responding in CUD.

**Electronic supplementary material:**

The online version of this article (10.1007/s00213-019-05330-z) contains supplementary material, which is available to authorized users.

## Introduction

Cocaine addiction is a global health problem that contributes to major economic and health burdens and is difficult to treat (Degenhardt et al. 2014). Although the initial positive reinforcing effects of cocaine are mediated by dopaminergic neurotransmission in the mesolimbic dopaminergic system, subsequent drug-seeking is guided by conditioning processes in a wider neural network (Everitt and Robbins 2005). Instrumental learning paradigms have provided a theoretical framework of impaired behavioural control for drug addiction (Everitt and Robbins 2005, 2016), as well as other psychiatric disorders (Robbins et al. 2012; Heinz et al. 2016). Instrumental learning is thought to be regulated by two distinct systems, namely the goal-directed and habit systems (Adams and Dickinson 1981). The goal-directed system, which is subserved by frontostriatal regions (Valentin et al. 2007; Tanaka et al. 2008; de Wit et al. 2009), controls voluntary instrumental behaviour by evaluating the potential consequences of actions. The habit system, which is subserved by corticostriatal circuits (Tricomi et al. 2009; Brovelli et al. 2011; de Wit et al. 2012; Zwosta et al. 2018), regulates automatic impulses in response to stimulus–response associations that have been formed over repeated experiences. Both systems are needed in everyday life, and optimal behavioural performance has been shown to require a balance between the joint regulation of these two systems (Balleine and O’Doherty 2010). A growing body of literature suggests that drug addiction develops through drug-induced disruption in corticostriatal subsystems that underlie these learning processes (Nelson and Killcross 2006; Belin and Everitt 2008; Gourley et al. 2013; Corbit et al. 2014). In most cases, drug-taking is initiated in a recreational setting and used in a goal-directed manner to experience pleasure. However, prolonged drug use in the same context may become habitual. As such, the initiation of drug-taking becomes triggered by environmental cues, irrespective of whether the experience of the drug is pleasurable (Miles et al. 2003; Vanderschuren and Everitt 2004). At the final stage of addiction, drug-taking habits predominate and may even continue in spite of harmful consequences (Everitt and Robbins 2005, 2016). It has been suggested that when habits spiral out of control, drug seeking is characterised by a failure to revert control toward the goal-directed system when the situational demands require it and become compulsive (Ersche et al. 2012).

A classic task to assess the balance between goal-directed and habit learning is the slips-of-action task (de Wit et al. 2007), which is based on an outcome devaluation paradigm to model the transition between behaviours that are initiated when obtaining reward and responses to a previously learned stimulus–response association. The extent to which participants maintain their previously learned behaviour despite outcome devaluation is considered an index of habit. Chronic cocaine and alcohol users (Sjoerds et al. 2013; Ersche et al. 2016), but not chronic tobacco smokers (Luijten et al. 2019), have been shown to develop a predominance of habits on this task, but the nature of their bias remains unclear. It has been hypothesised that either difficulties with goal-directed learning facilitate the transition of control from the goal-directed toward the habit system or an *augmented* control by the habit system results in habit predominance (Robbins and Costa 2017; Vandaele and Janak 2018). Whilst the bulk of prior work has focused on cocaine’s influence on the transition of control from the goal-directed to the habit system, less attention has been given to its influence on goal-directed learning.

Reinforcement learning algorithms implement learning and action selection in response to motivationally relevant reinforcement (Russell and Norvig 1995; Sutton and Barto 1998). Basic parameters in a typical reinforcement learning model are learning rate (α) and reinforcement sensitivity (also known as choice inverse temperature, *β*). *Learning rates* modulate the extent to which information is learnt, with higher rates indicating that feedback is integrated more rapidly in order to inform future choices. *Reinforcement sensitivity* regulates the influence of associative strength during action selection, with higher sensitivity reflecting a greater impact of action values on choices. Such reinforcement learning models can be fitted to the observed behaviour, yielding estimates of the model’s parameters, and different models can be compared, allowing learning to be investigated in a hypothesis-driven manner (Daw 2011). One additional parameter relevant to drug addiction is the tendency for *perseverative responding* (sometimes termed ‘stickiness’). As chronic cocaine use has been associated with profound reversal learning deficits in both animals and humans exposed to cocaine (Schoenbaum et al. 2004; Calu et al. 2007; Ersche et al. 2008, 2011), it is possible that inflexible contingency evaluations may also contribute to their learning deficits.

In the present study, we apply a hierarchical Bayesian approach to previously published data using the slips-of-action task in both healthy volunteers and patients with cocaine use disorder (CUD) (Ersche et al. 2016). We hypothesise that overall poor learning performance in CUD patients can be explained by abnormalities in at least one of the following parameters: learning rate, reinforcement sensitivity, perseveration and extinction. The latter parameter, extinction, was included in the model in light of its relevance for subsequent habit learning. *Extinction* describes the ability to learn from non-rewarding events. Given that habit formation has also been described in terms of behavioural autonomy (Dickinson 1985), it is conceivable that habits form more easily in individuals who are resistant to extinction. We further predict that white matter integrity of the goal-directed system is required for successful action-outcome learning and that deficiencies would facilitate the formation of habitual responding.

## Methods

### Sample

Fifty-five healthy control volunteers (94.3% male) and 70 patients with CUD (90.3% male) were recruited for the study. Full details of the sample can be found elsewhere (Ersche et al. 2016). All CUD patients were recruited from the local community and satisfied the DSM-IV criteria for cocaine dependence (American Psychiatric Association 2013). Forty-eight CUD patients also met DSM-IV criteria for opiate dependence, 25 for cannabis dependence and five for alcohol dependence. Twenty-six CUD patients were prescribed methadone (mean dose 48.7 ml, SD ± 18.0) and 14 were prescribed buprenorphine (mean dose 7.2 ml, SD ± 4.8). Although significantly more CUD patients (94%) reported smoking tobacco compared with control volunteers (11%) (Fisher’s *p* < 0.001), nicotine dependence was not assessed using the DSM-IV criteria. CUD patients had been using cocaine for an average of 16 years (7.7 ± SD) and were at the time of the study all active users of the drug, as verified by urine screen. Two CUD patients were excluded due to incomplete data sets. Healthy control volunteers were partly recruited by advertisement and partly from the BioResource volunteer panel (www.cambridgebioresource.group.cam.ac.uk). None of the healthy volunteers had a history of drug or alcohol dependence. The following exclusion criteria applied to all participants: no history of neurological or psychotic disorders, no history of a traumatic brain injury, no acute alcohol intoxication (as verified by breath test) and insufficient English proficiency. All volunteers consented in writing and were screened for current psychiatric disorders using the Mini-International Neuropsychiatric Inventory (Sheehan et al. 1998). Psychopathology in drug users was further evaluated using the Structured Clinical Interview for DSM-IV (First et al. 2002). All participants completed the National Adult Reading Test (NART) (Nelson 1982) to provide an estimate of verbal IQ and the Alcohol Use Disorders Identification Test (AUDIT) (Saunders et al. 1993) to evaluate the pattern of alcohol intake.

### Slips-of-action task

**Fig. 1:**
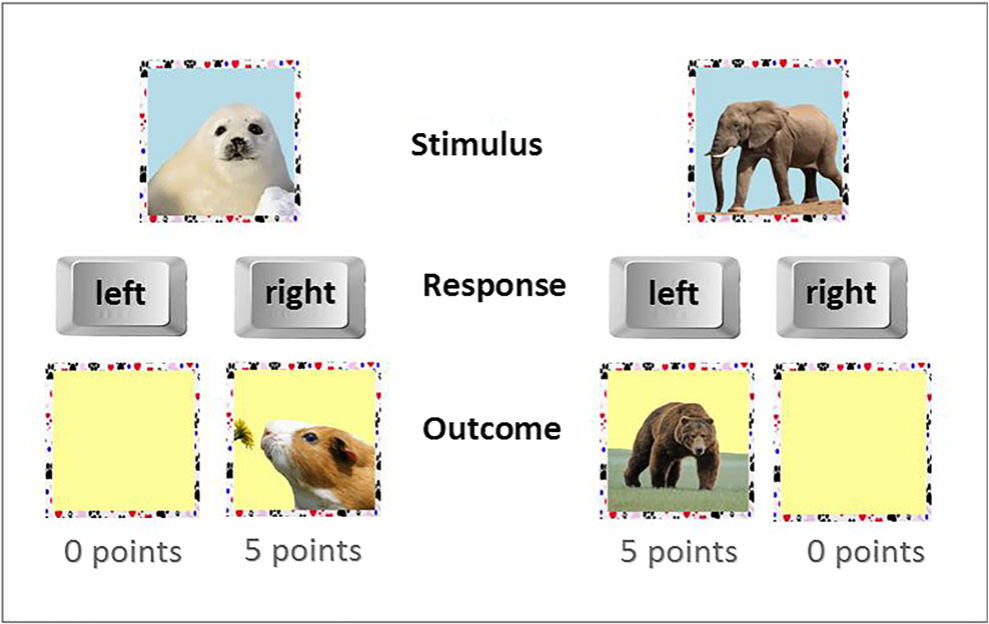
Outline of the appetitive discrimination learning task. Participants were required to learn by trial and error which response associated with an animal picture gained them points. Feedback was provided by a picture of another animal coupled with either a number of points or an empty box with no points

Details of the task are reported elsewhere (Ersche et al. 2016). In brief, in the first part of the task, participants complete an appetitive discrimination task in which they learn over 96 trials the associations between a response (left or right button press) and a rewarding outcome (gaining points or no points). On each trial, participants were presented with one of six animal pictures and were instructed to learn by trial-and-error which button to press in order to gain points (see Fig. 1). Feedback was provided immediately. The rewards were delivered deterministically, i.e. there is only one correct response for each stimulus. Correct responses were recorded as an index of learning from positive reinforcement.

Completion of the first phase led to the second phase, in which participants were instructed to select the correct response for each animal picture as quickly as possible. However, some outcomes were devalued such that participants were told that responses for certain animal pictures were no longer valuable, and they should not be selected (i.e. participants had to withhold their response). No feedback was provided during this phase, which consisted of nine 12-trial blocks, which at the start of each block, informed participants about the devalued outcomes. Responses toward devalued animal pictures are considered ‘slips of actions’ and have been suggested to reflect habitual control (de Wit et al. 2007, 2009). We calculated a ‘habit bias’, based on responding to devalued stimuli minus responding to valued stimuli. Participants, who respond in a goal-directed fashion, will follow the instruction to only respond to the stimuli that carry a value. However, sometimes, they may fail to do so, making a ‘slips-of-action’ such that they respond to devalued stimuli although they do not carry any more points. For these participants, their habit bias will be low or even negative. By contrast, participants who respond in a habitual manner will not make this distinction between valued and devalued outcome, as they continue responding equally often to devalued and the value stimuli, making frequent slips of action, so that their habit bias (or slips-of-action score) is likely to be high and close to zero.

### Statistical analysis and computational modelling

#### Demographic and behavioural data

Data were analysed using the Statistical Package for the Social Sciences, v.22 (SPSS, Ltd.). Group differences regarding demographics and fractional anisotropy (FA) values of the goal directed, as well as the habit system pathway were analysed using independent samples *t* tests. The white matter tracts between the medial orbitofrontal cortex and the anterior part of the caudate nucleus have previously been shown to underlie goal-directed control, whereas the tracts between the posterior putamen and the premotor cortex is thought to subserve habit control (de Wit et al. 2012). To determine the learning parameters that subsequently affected habitual responding, we performed a stepwise regression model, in which we included the three relevant learning parameters of the model (learning rate, reinforcement sensitivity, perseveration), group status and white matter integrity between the medial orbitofrontal cortex and the anterior caudate nucleus (as reflected by FA values). We also calculated Pearson’s correlation coefficients to evaluate putative relationships between these learning parameters, demographic variables and the duration of cocaine use. To address the question as to whether proneness to habits in CUD patients is due to deficits in goal-directed learning, we fitted an ANCOVA model and included the parameter learning rate as a covariate. All statistical tests were two-tailed and significance levels were set at 0.05.

#### Reinforcement learning algorithm

We fitted trial-by-trial performance on the appetitive learning phase with a delta rule to model the choice selection process. Since there are two possible responses for each stimulus (i.e. ‘respond right’ and ‘respond left’), the associative strength for the chosen stimulus–response pairing on a given trial, *V*_*t*_, was updated, using the following algorithm:

$$ {V}_{t+1}={V}_t+\alpha \left({R}_t-{V}_t\right) $$

When a particular response is positively reinforced, the associative strength for the stimulus–response association increases. This associative strength for each stimulus–response pairing is updated on a trial-by-trial basis via prediction errors that represent discrepancies between expected outcome, *V*_*t*_, and actual outcome, *R*_*t*_. Larger prediction errors thus lead to greater changes in associative strength. The sensitivity to this prediction error is regulated by the free parameter, *α*. Higher *α* represents increased sensitivity to prediction errors, resulting in quicker updating of associative strengths and enhanced learning.

There is evidence for differential neural processing of reward and non-reward (Kim et al. 2006), suggesting that these two processes may be dissociable. To account for this possible distinction, we tested two classes of computational models. In one class, we fractionated *α* based on the context. Trials that are positively reinforced were updated by an appetitive learning rate, *α*^*rew*^, whereas trials that were not reinforced were regulated by an extinction rate, *α*^*ext*^. (Increases in *α*^*rew*^ would indicate increased learning from reinforcement, and increases in *α*^*ext*^ similarly from non-reinforcement.) In a second class, we used a single *α* value, termed learning rate, to modulate prediction errors irrespective of outcome. We also allowed for the fact that a subject may ‘stick with’ or perseverate to the response that they selected on the previous trial. For trial *t* and response *k*, we defined *C*^*k*^_*t*_ to be 1 if the subject chose response *k* on the previous trial (trial *t* − 1) and 0 otherwise. We then defined a perseveration parameter *τ* through which a putative tendency to perseverate influenced behaviour, alongside the reinforcement learning process.

Associative strengths and perseverative tendencies were then used to select actions. This process followed a softmax rule, according to the following equation:

$$ p\left(i,t\right)=\frac{e^{\beta {V}_t^i+\tau {C}_t^i}}{\sum_{k=1}^n{e}^{\beta {V}_t^k+\tau {C}_t^k}} $$

This softmax equation gives the model’s predicted probability of a given choice *i* on a given trial *t*. Associative strengths (calculated as above) drive choices, and the degree to which they influence the final choice is determined by the reinforcement sensitivity parameter *β*. A tendency to perseverate can also influence choice, and the degree to which this happens is determined by the perseveration parameter *τ*. As outlined in Table 1, there are four possible free parameters that were modelled: learning rate, extinction rate, reinforcement sensitivity and perseveration.

**Table 1:** **Summary of the reinforcement learning models tested.** Several models with different parameter combinations were assessed via bridge sampling. We show the included posterior probabilities for each model, i.e. the probability of each model given the data (and given that they were equiprobable before the data). Models were ranked accordingly and we found that the best-fit model used three parameters: learning rate, reinforcement sensitivity and perseveration. We have also included log Bayes factors for comparisons between the ranked models. According to the criteria of Kass and Raftery (1995), there was overwhelming evidence that the top two ranked models were superior to all other models. Though the difference between the top two models was marginal, we have selected the model that was more likely, which was also the more parsimonious of the two. [Note: Logs are natural logarithms unless stated.]

Free parameters				Model selection				
Learning rate^a^	Extinction rate, *α*^*ext*^	Reinforcement sensitivity, *β*	Perseveration, *τ*	Log marginal likelihood	Log posterior *p*(model)	Posterior *p*(model)	Log_10_ Bayes factor (relative to next-ranked model)	Ranking
✓		✓	✓	− 6718.8	− 0.578	0.561	0.106	1
✓	✓	✓	✓	− 6719.0	− 0.823	0.439	18.03	2
✓	✓	✓		− 6760.5	− 42.33	0	0.407	3
✓		✓		− 6761.5	− 43.27	0	140.71	4
✓	✓		✓	− 7085.5	− 367.27	0	20.04	5
✓	✓			− 7131.6	− 416.40	0	492.78	6
				− 8266.3	− 1548.06	0	N/A	7 ^b^

^a^For some models, the learning rates were fractionated into learning from reward (*α*^*rew*^) or non-reward (i.e. extinction rate, *α*^*ext*^), as shown. If extinction rate is not defined in the model, then the learning rate should encompass learning from both reward and non-reward (*α*).

^b^To verify that these results were not spurious findings, we included a random choice model, which assumes that choices were selected at random (*p* = 0.5 for each of the two possible responses). Our results suggest that all tested models fit the data better than the random choice model.

The task design involved an explicit instruction of a different task context and different performance rules in the second phase, gave no feedback, and successful performance relies on explicit representation of instrumental value as instructed. These limitations prevented accurate trial-by-trial modelling of behaviour from the second phase within this model. An additional confirmatory model, representing goal-directed action and habit learning explicitly, was therefore used to check the effects of outcome devaluation (see below).

#### Parameter estimation

Free parameters from reinforcement learning algorithms were estimated using a hierarchical Bayesian approach. This approach produces a posterior distribution for all parameters of interest. We defined prior distributions for all parameters. The learning rate parameters alpha (*α*, *α*^*rew*^, *α*^*ext*^), which have the range [0, 1], were given a prior beta (1.1, 1.1) distribution. Reinforcement sensitivity, *β*, was given a prior gamma (4.82, 0.88) distribution (Gershman 2016). Perseveration, *τ*, was given a normal (0, 1) prior; perseverative parameters can be negative, indicating anti-perseveration (switching behaviour) (Christakou et al. 2013).

At the top level of the hierarchy, for each parameter, we defined a separate distribution for each group (CUD and controls). These were the primary measures of interest. Each individual subject’s parameter was drawn from a distribution about their group-level parameter, with the assumption that individual subjects’ differences from their group mean had a normal distribution with mean 0 and a parameter-specific standard deviation (necessarily positive). For *α* and *τ*, this standard deviation was drawn from a prior half-normal (0, 0.17) distribution. For *β*, the standard deviation of inter-subject variability was drawn from a prior half-normal (0, 2) distribution. Final subject-specific parameters were bounded as follows: *α* ∈ [0,1]; *β* ∈ [0,+∞]; *τ* ∈ [−∞, +∞]. These final subject-specific parameters were then used in a reinforcement learning model, whose output was the probability of selecting each of the two actions on any given trial. The model was fitted (yielding posterior distributions for each parameter) by fitting these probabilities (arbitrarily, the probability of choosing the right-hand response) to actual choices (did the subject choose the right-hand response?).

We conducted the Bayesian analysis in RStan (Carpenter et al. 2017), which uses a Markov chain Monte Carlo method to sample from posterior distributions of parameters. We used R version 3.3.3–3.6.0 and RStan version 2.17.2–2.18.2. We simulated 8 parallel chains, each with 8000 iterations. We assessed the convergence of the simulations by checking the potential scale reduction factor measure, R-hat (Gelman et al. 2013). R-hat values of 1 indicate perfect convergence. We used a stringent cut-off of < 1.1 as an indicator for sufficient convergence of the simulations (Brooks and Gelman 1998). Starting each simulation runs from a different point, with automatic measurement of convergence, is an important check for simulation reliability, and is an intrinsic part of Stan. Primary values of interests were posterior distributions of the group difference (CUD—control) for each free parameter. Measures of dispersion of posterior distributions were denoted as 95% highest density intervals (HDI). Given the assumptions (priors, model) and data, there is a 95% probability that the true value lies within the 95% HDI. An HDI of the group difference that does not overlap with zero indicates credible group differences.

#### Model selection

As shown in Table 1, several variants of the models were tested against each other. The best model was determined using bridge sampling (Gronau et al. 2017), which estimates model fit. The bridge sampling procedure computes the probability of the observed data given the model of interest, the marginal likelihood *P*(*D* | *M*), which encompasses both the probability of the data given specific values of the model’s parameters, the likelihood *P*(*D* | *θ*, *M*) (is there a good fit?) and the prior probability of the parameter values given the model, *P*(*θ* | *M*) (thus encapsulating a penalty for over-complex models; Occam’s razor). The marginal likelihoods *P*(*D* | *M*_*i*_) can be combined with prior model probabilities *P* (*M*_*i*_) to obtain posterior model probabilities *P* (*M*_*i*_ | *D*). We report posterior probabilities for the models, which indicate evidence for the model; a higher probability indicates a better model. Additionally, we also report the log Bayes factor as a second indicator of model evidence, Bayes factors being ratios of marginal likelihoods of a pair of models. We assumed models were equiprobable a priori.

#### Confirmatory modelling of goal-directed action and habitual responding

To analyse more directly the question of whether the balance between goal-directed and habitual systems was altered in the CUD group, as assessed by the outcome devaluation procedure, we developed and simulated a full two-system model of instrumental learning as an additional check. This model implemented outcome devaluation via instantaneous instruction (see Supplementary Material). The behavioural task (Ersche et al. 2016) was incompletely specified for this fuller instrumental model in some respects, in that it did not permit independent evaluation of the learning rate for habit and goal-directed systems, though it permitted evaluation of the relative expression of those two systems via the outcome devaluation phase. The behavioural task was also ambiguous as to whether the framing of the task was likely to have allowed further S–R habit learning (as distinct from expression) during the outcome devaluation phase, given that the response instructions were altered substantially in this phase; we therefore tested models with and without S–R learning during this test phase (‘habit learning at test’, HLAT, or ‘no habit learning at test’, NHLAT; see Supplementary Material), with the caveat that the HLAT model had the potential to confound the effects of outcome devaluation and extinction in the measurement of learning rate.

### Neuroimaging data

To address the critical question of whether abnormal learning performance is associated with variations in frontostriatal connectivity, we obtained neuroimaging data from almost 70% of our participants (44 controls, 44 CUD). The selection of this subgroup was based on MRI-suitability and availability for the acquisition of the scan. The subgroup was representative of the entire sample, as no significant group differences in their demographic profiles were identified.

#### MRI data acquisition, pre-processing and ROI generation

The brain scans were acquired at the Wolfson Brain Imaging Centre, University of Cambridge, UK. T1-weighted MRI scans were acquired at by T3 Siemens Magenetom Tim Trio scanner (www.medical.siemens.com) using a magnetization-prepared rapid acquisition gradient-echo (MPRAGE) sequence (176 slices of 1 mm thickness, TR = 2300 ms, TE = 2.98 ms, TI = 900 ms, flip angle = 9°, FOV = 240 × 256). One CUD scan was removed due to excessive movement. All images were quality controlled by radiological screening. The MPRAGE images were processed using the recon-all Freesurfer (v5.3.0, recon-all, v 1.379.2.73) pipeline to generate individually labelled brains using the standard subcortical segmentation and Destrieux atlas surface parcellations. Two regions of interest (ROIs) were created in both the left and right hemispheres: the combined caudate and nucleus accumbens, the medial orbitofrontal cortex, as well as the premotor cortex (BA6) (thresholded version) and posterior putamen (defined as the putamen for *y* ≤ 2 mm in MNI space (see de Wit et al. 2012)). A mask was created in MNI space for *y* > 2 mm. The inverse MNI transform for each individual was applied to the mask to put it in native conformed space, which was then used to split the putamen into posterior and anterior portions. In addition, two exclusion masks were created comprising each hemisphere and all ventricles. All ROIs were transformed into diffusion-weighted imaging data (DWI) space for the subsequent tractography analysis.

#### DWI data acquisition and pre-processing

Due to excessive movement, four scans had to be excluded from the analysis (1 control, 3 CUD). DWI volumes were successfully acquired from 84 participants (43 controls, 41 CUD) using 3T Siemens Magenetom Tim. All DWI scans were acquired within the same session as the MPRAGE data set. Sequence details were as follows: TR = 7800 ms, TE = 90 ms, 63 slices of 2 mm thickness, 96 × 96 in-plane matrix and FOV = 192 × 192 mm. DWI data were acquired with a 63 direction encoding scheme. These 63 volumes were acquired with a *b* value of 1000 s/mm^2^ following an initial volume with a *b* value of 0 s/mm^2^.

The DWI images were processed using the standard FSL (FMRIB Software Library; Release 5.0.6) tractography pipeline. First, eddy correct was performed to correct head motion and distortion, and align the series to the b0 image. Next, a brain mask was created by applying bet to the b0 image. Then, diffusion parameters were estimated using bedpostX. BedpostX uses a Bayesian framework to estimate local probability density functions on the parameters of an automatic relevance detection multicompartment model. In this case, two fibres per voxel were modelled. Following bedpostX, probabilistic tractography was applied to the diffusion parameters using probtrackx2. Probtrackx2 computed streamlines by repeatedly generating connectivity distributions from voxels in seed ROIs. The default settings of 5000 samples per voxel and 0.2 curvature threshold were used. Analyses were performed from seed ROIs to waypoint targets in each hemisphere separately with an exclusion mask defined for each analysis comprising the combined contralateral hemisphere and ventricles. The first seed-target path interrogated was caudate and nucleus accumbens to medial orbitofrontal cortex, and the second seed-target path interrogated was posterior putamen to the premotor cortex, which made a total of four analyses per participant. Each analysis generated a waytotal, which is the number of tracts surviving the inclusion and exclusion criteria. Each participant’s waytotals were normalised by the individual seed ROI volumes (× 5000) to produce single measures of tract strength between the seed and the target.

In addition to the waytotal, each tractography analysis produced a connectivity distribution path. A summary group path distribution was produced to illustrate each tract. Each individual path was thresholded above 5% or 10 hits, whichever was the higher value. These paths were then transformed into MNI space using a non-linear warp and a mean path created. Individual seed and target regions were also transformed into MNI space using the combined Freesurfer to diffusion space affine transformation and the non-linear diffusion to MNI space warp. A summary binary region of interest was created representing the path from the combined caudate and nucleus accumbens to medial orbitofrontal cortex. The ROI comprised voxels containing thresholded paths from at least half the participants.

FA maps were created using FSL’s dtifit and were then processed according to the standard tract-based spatial statistics (TBSS) pipeline to create a 4D volume containing each participant’s skeletonised FA image. Mean FA values were calculated for each participant within the group ROI from each tractography path (anterior caudate to medical OFC and putamen to premotor cortex) and imported into SPSS for post hoc analyses.

## Results

### Group characteristics

As reported previously, the groups were matched in terms of age, gender and alcohol intake (all *p*s < 0.05) but differed significantly in terms of verbal IQ (*t*_120_ = 8.8 *p* = 0.019). However, only in control volunteers IQ scores were correlated with learning rate (*r* = .29, *p* = 0.034) and reinforcement sensitivity (*r* = .30, *p* = 0.029), but not in CUD patients (both *p* > 0.1). We also found that in CUD patients, the duration of cocaine use correlated significantly with the degree of response perseveration (*r* = .29, *p* = 0.014), but prolonged cocaine use showed no relationship with either learning rate (*r* = − .14, *p* = 0.254) or reinforcement sensitivity (*r* = − .19, *p* = 0.118).

### Instrumental learning performance

**Fig. 2:**
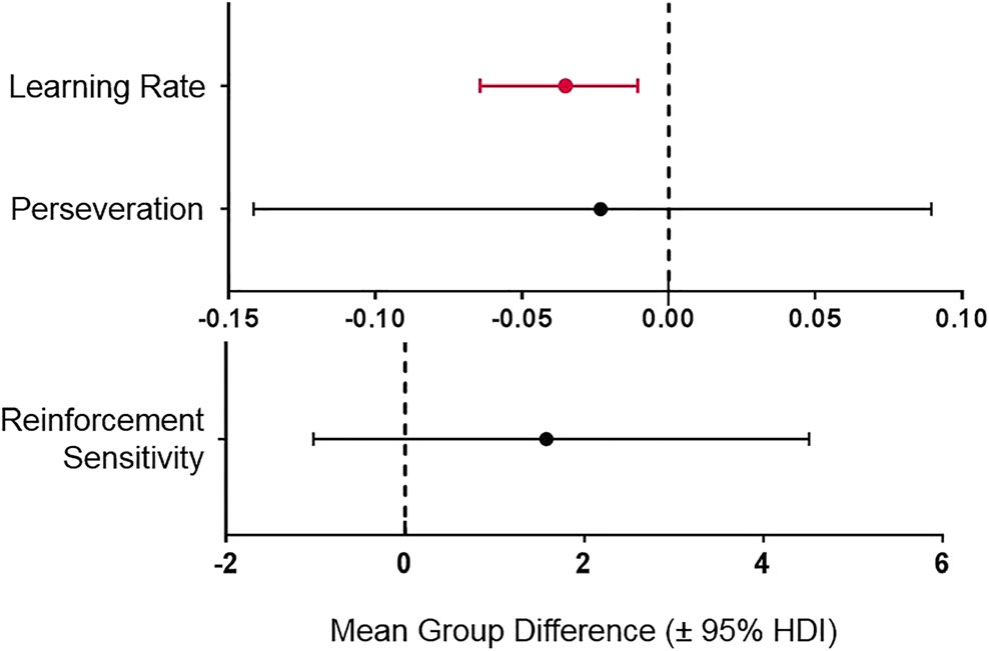
The mean group differences of the posterior distributions for each learning parameter in the model. Parameters that have group differences (indicated in red) have 95% highest density intervals that do not overlap zero. Compared with healthy control volunteers, patients with CUD show a reduced learning rate. Both mean differences in reinforcement sensitivity and perseveration did overlap with zero. (Note: the reinforcement sensitivity parameter is placed on a different axis due to scale differences.)

As shown in Table 1, the winning model contained three parameters: a single learning rate, reinforcement sensitivity and perseveration (‘stickiness’). Relative to healthy control volunteers, CUD patients demonstrated reduced learning rates (see Fig. 2; posterior probability of non-zero difference, pNZ = 0.999, posterior mean difference, *d* = − 0.035, 95% HDI = − 0.064 to − 0.010). There were no group differences for reinforcement sensitivity (pNZ = 0.69, *d* = 1.58, 95% HDI = − 1.02 to 4.51) or perseverative responding (pNZ = 0.367, *d* = − 0.02, 95% HDI = − 0.141 to 0.089). Across subjects, learning rate and reinforcement sensitivity were correlated but other parameters were not (Supplementary Material, Fig. S1) Convergence of the winning model was very good; all parameters and contrasts had R-hat values of less than 1.1 (maximum R-hat = 1.03).

In light of the high prevalence of co-morbid opiate use in cocaine addiction, we also subdivided the CUD sample into CUD participants with (*n* = 22) and without co-morbid opiate dependence (*n* = 48) and fitted the winning model with data of these two subgroups. As shown in Table S1**,** the two subgroups did not differ on any performance parameter.

In the additional model examining goal-directed actions and habits across both task phases, whether or not S–R learning was assumed to occur during the test (second) phase influenced the sign of the difference in learning rate observed in this two-system model (measured by variational Bayes approximation see Supplementary Material, Table S4), rendering interpretation of learning rates difficult. In the NHLAT model, the CUD group showed lower learning rates than controls; this is entirely consistent with the lower learning rates found via the main computational model confined to the first phase of the task (since in that model and the NHLAT model, learning rates were only measured during the initial learning phase). In the HLAT model, learning rates were higher in the CUD group; this likely reflects a confound between measuring the impact of outcome devaluation and measuring extinction in the second phase, altering the estimates of learning rates.

However, other aspects of the additional two-system models were consistent. Both the NHLAT and HLAT models showed a reduced impact of the goal-directed action system in the CUD group, no difference in the impact of the habitual system and a somewhat greater tendency to perseverate (or lesser tendency to switch response) in the CUD group (Supplementary Material, Table S4). These results were supported by a full Markov chain Monte Carlo fit (see Supplementary Material, Table S5), showing reductions in the impact of the goal-directed system and an increase in perseveration in the CUD group, across both the NHLAT and the HLAT model, without changes in the impact of the habitual system (or in these simulations, the learning rate parameter). These results are therefore consistent with a reduction in the relative efficacy of goal-directed action and an increase in the relative (if not absolute) efficacy of habitual learning in patients with CUD. Moreover, since the goal-directed system was consistently less effective in CUD patients, in addition to and independent of changes in learning rate, the results of both the NHLAT and HLAT models support the conclusion that excessive dominance of the habit system (due to impaired goal-directed action) in CUD patients is not explicable purely in terms of changes in learning rates.

### Relationships between learning performance and white matter integrity

**Fig. 3:**
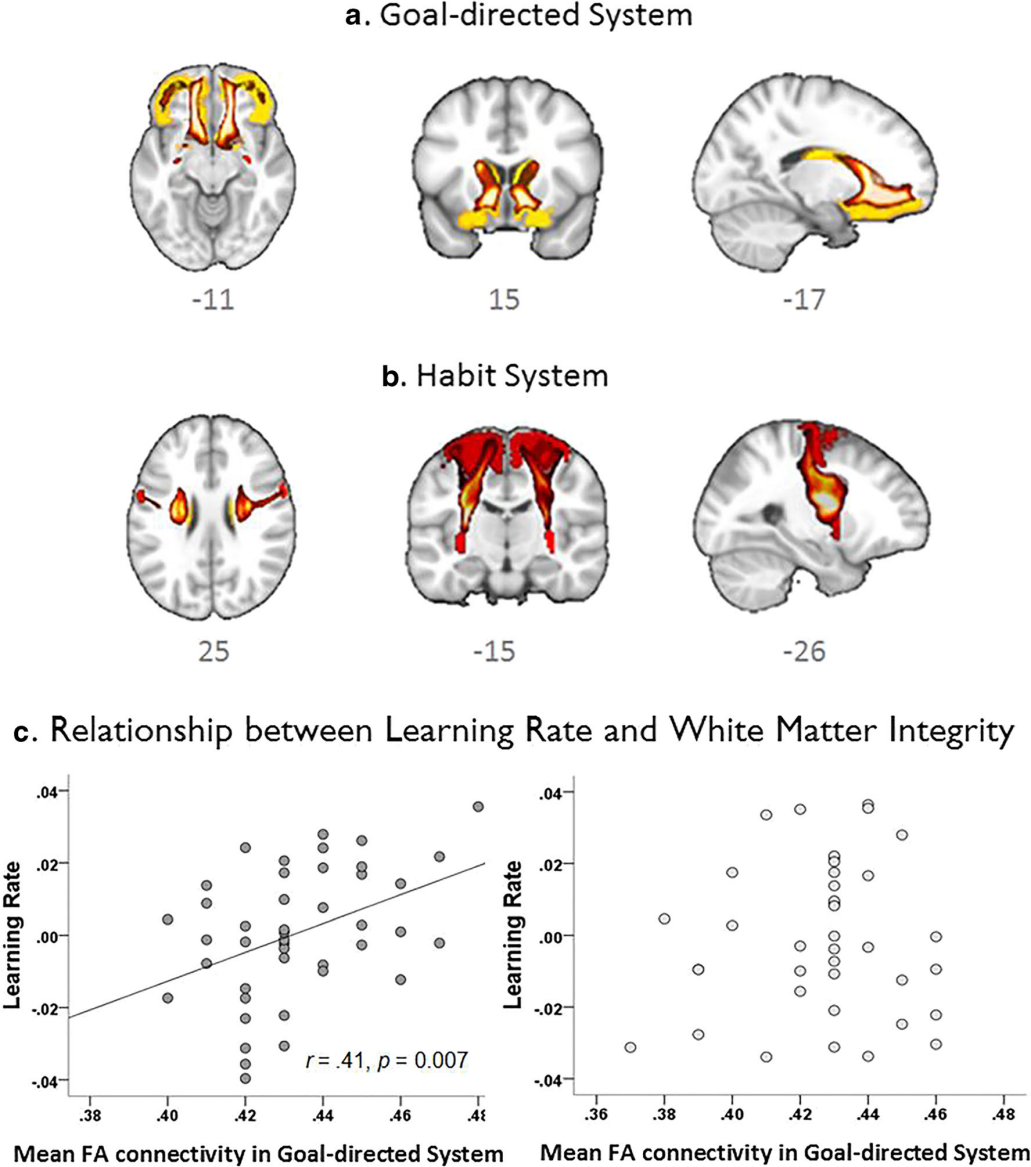
Structural connectivity of mean fractional anisotropy (FA) between brain regions involved in **a** the goal-directed system, which has been linked with interactions between the medial prefrontal cortex, the anterior caudate nucleus and ventral parts of the striatum, and **b** the habit system, which depends on interactions between pre-motor cortex (BA6) and the posterior putamen. **c** Scatter plot depicting the significant relationships in healthy control volunteers between learning rates and mean FA values within the neural pathway that has been suggested to underlie goal-directed learning*.* Scatter plot showing the lack of such a relationship in CUD patients

We compared the two groups with respect to white matter integrity, as reflected by fractional anisotropy (FA) values, within both the goal-directed and the habit pathways. Whilst FA values between the anterior caudate–medial OFC (goal-directed) pathways did not significantly differ between CUD patients and control volunteers (*t*_81_ = 1.57, *p* = 0.122), we identified significant group differences in white matter integrity in the putamen-premotor cortex (habit) pathway as FA in the CUD group was significantly reduced compared with controls (*t*_81_ = 2.19, *p* = 0.031). We first correlated, separately for each group, the learning rates with mean FA values of the goal-directed pathway and then the slips-of-action scores with mean FA values in the habit pathway (see Fig. 3). Learning rates showed a positive correlation only in control volunteers (*r* = .406, *p* = .007), but not in CUD patients (*r* = .070, *p* = .668), whereas the slips-of-action score was not correlated with the FA values in either group (controls: *r* = − .25, CUD: *r* = .05; both *p* > 0.1).

To further examine the extent to which learning performance accounted for individual variation in habitual responding, we employed a stepwise regression model analysing habit bias (slips-of-action) scores. The model revealed that group status accounted for 12% of the variance in habitual responding (*β*_group_ = 0.362, *R*^2^ = 0.12, *F*_1,121_ = 18.24,*p* < 0.001). When reinforcement sensitivity was entered in the model, about a quarter of the variance (25%) was explained by the two factors (*β*_group_ = 0.358, *β*_reinf_ = − 0.355, *R*^2^ = 0.25, *F*_2,120_ = 20.77,*p* < 0.001); learning rate and perseveration had no explanatory value (i.e. the addition of these parameters did not significantly improve the model). When we subsequently entered the neural correlates of the goal-directed pathway, which were available in 70% of the sample, the results did not change. In this smaller sample, group status explained 17% of the variance (*β*_group_ = 0.425, *R*^2^ = 0.17, *F*_1,81_ = 17.82,*p* < 0.001) and, together with reinforcement sensitivity, explained 30% of the variance of habitual responding (*β*_group_ = 0.403, *β*_reinf_ = − 0.365, *R*^2^ = 0.30, *F*_2,80_ = 18.23,*p* < 0.001), suggesting that the strong habit bias in CUD was not fully explained by the deficits in discrimination learning. This was further supported by the fact that the strong habit bias in CUD was also seen when the learning rate was included as a covariate in the analysis (*F*_1,120_ = 20.2, *p* < 0.001). Given that the groups also differed in white matter integrity in the habit pathway, we added FA values of the putamen-premotor (habit) pathway as a second covariate in the ANCOVA model, but this did not affect the significant habit bias in CUD patients (*F*_1,79_ = 16.9, *p* < 0.001).

Although the groups did not differ with respect to FA within the goal-directed pathway (*t*_81_ = 1.57, *p* = 0.122), we aimed to evaluate the putative relationships between the three learning parameters and FA. We calculated Pearson’s correlation coefficients, which revealed relationships between the learning rate (*r* = .41, *p* = 0.007) and reinforcement sensitivity (*r* = .34, *p* = 0.026) only in the control volunteers but not in CUD patients (both *p* > 0.5). Using Fisher’s transform, we found that the correlations between learning rate and FA were only marginally different from each other (*Z* = 1.56, *p* = 0.059; one-tailed).

## Discussion

Drug addiction has been described as a disorder of learning and memory (Hyman 2005), where behavioural choices become biased toward highly reinforcing drug rewards which persist even if the anticipated rewarding outcome does not materialise. Here we deconstructed the process of appetitive discrimination learning in a non-drug related context in both healthy control participants and patients with CUD using a computational modelling approach, which yielded two important findings. Firstly, we demonstrated that CUD patients exhibit significant deficits in reinforcement learning as reflected by a reduced learning rate in a simple RL model, possibly indicating problems with making accurate reward predictions and/or updating these predictions based on feedback. Secondly, we demonstrated that the reduced learning rate in CUD patients did not, however, fully explain their proneness for stimulus–response habits during instrumental learning. Habitual response tendencies, as measured by reward devaluation, were partly explained by the diagnosis of CUD and individual variation in reinforcement sensitivity but were not sufficiently explained by deficits in learning. These conclusions were supported by additional analyses across discrimination and devaluation phases using a two-system model representing goal-directed action and habit learning, which showed a reduced impact of the goal-directed system in CUD patients. Changes in learning rate were not sufficient to explain the relative predominance of the habit system in CUD patients.

### Deficits in learning from positive feedback impair appetitive discrimination learning in CUD

Our findings are strikingly consistent with previous reports in both animals and humans, suggesting that chronic cocaine use is associated with deficits in the processing of positive feedback (Lucantonio et al. 2015; Morie et al. 2016; Takahashi et al. 2016; Strickland et al. 2016). By changing the neuronal signalling patterns, chronic cocaine use has been suggested to alter the encoding of outcome information such as value, timing and size of the outcome, thereby hampering predictions about the consequences of one’s actions (Takahashi et al. 2019). Our findings are also consistent with work by Kanen et al. (this issue), who also identified in another sample of stimulant-addicted individuals a reduced learning rate from positive feedback. It is noteworthy that those authors further showed that the learning deficits were amenable to dopaminergic modulation, thus supporting the notion of mediation via alterations in the firing patterns of dopamine neurons. The nature of the hypothesised cocaine-induced neuroadaptive changes of appetitive learning may also explain why we did not find changes in white matter integrity within the goal-directed pathway. We only found a lack of the normal relationship between learning from positive feedback and structural integrity in CUD patients but did not find significant structural alterations. It must also be emphasised that CUD patients’ ability to learn from positive feedback was not entirely impaired. All participants in the study were able to learn the stimulus-reward associations, but CUD patients learned them less well than healthy control participants. Their ability to learn from positive feedback also stands in stark contrast from that of learning from negative or punishing feedback, which has been repeatedly shown to be severely impaired in CUD patients (Tanabe et al. 2013; Hester et al. 2013). Such an imbalance in the ability to process reinforcing feedback has important ramifying effects on patients’ decisions and behavioural choices and therefore should be recognised as a treatment need.

### Diagnosis of CUD and variation in reinforcement sensitivity partly explain habit bias

The mechanism that renders CUD patients prone to developing stimulus–response habits is not fully understood. The weaker white matter integrity in the habit pathway in CUD patients was, however, unrelated to behaviour, suggesting that the increased habit bias cannot simply be attributed to structural variations. However, it has been previously suggested that a strong habit bias could reflect a compensatory response to a weakened goal directed system (Robbins and Costa 2017; Vandaele and Janak 2018). Here we demonstrate that reduced learning rate in CUD patients does not account sufficiently for their proneness to form stimulus–response habits. Other psychiatric disorders, such as obsessive-compulsive disorder, exhibit a habit bias on this task alongside unimpaired discrimination learning (Gillan et al. 2011). It is conceivable that the regulatory balance between goal-directed or habitual control is disrupted in CUD patients, indicating a failure to revert control to the goal-directed system following a rule change. Alternatively, but not mutually exclusively, it is also possible that habitual control is generally more predominant in cocaine addiction. Whilst there is ample evidence showing failure of CUD patients to adjust cognitive or behavioural responses to changing situational demands (Lane et al. 1998; Verdejo-García and Pérez-García 2007; Ersche et al. 2008, 2011; McKim et al. 2016), far less research has addressed the predominance of the habit system.

Our data further indicate that one learning parameter in particular, reinforcement sensitivity, does seem to be involved in habit formation. This observation is not surprising given that habit learning in this study was assessed using a reward devaluation paradigm, which deliberately manipulates the value of the expected outcome of an instrumental response to make the outcome less desirable and the behavioural response less likely. If these manipulations, however, do not impact on performance, it may indicate that behaviour is not controlled by the anticipated consequences but by antecedent stimuli, or in other words, their behaviour has become habitual. Although reinforcement sensitivity values in this study did not differ between the groups, it is noteworthy that correct responses were reinforced by the points gained, which CUD patients may not have perceived as rewarding. Future research may thus need to evaluate whether the use of more reinforcing incentives such as monetary gain or the prospects of desirable benefits would be more appropriate for a reward devaluation paradigm than gaining points, possibly making devaluation more effective in inducing behavioural change.

### Neural substrates of appetitive discrimination learning

Our data also indicate that the diagnosis of CUD, rather than individual learning parameters, critically account for the facilitated transition from goal-directed to habitual responding. The diagnosis may thus reflect disorder-related changes within corticostriatal networks that subserve associative learning, which are likely to promote the devolution of control from the goal-directed to the habit system (Nelson and Killcross 2006; Takahashi et al. 2007). Cocaine addiction has been associated with numerous changes within dopaminergic pathways such as low D2 receptor density in the striatum and reduced orbitofrontal metabolism (Volkow et al. 1993), blunted stimulant-induced dopamine release (Martinez et al. 2007), reduced white matter integrity in the inferior frontal gyrus (Ersche et al. 2012) and altered cognitive responses to dopamine agonist challenges (Ersche et al. 2010). Loss of white matter integrity specifically in the inferior frontal gyrus might also play a role in disinhibited behaviour, whereas action selection is undermined by alterations in dopaminergic transmission. More research is warranted to investigate the neuromodulatory effects of specifically dopaminergic agents on associative learning. Work by Kanen et al. (this issue) already shows some promising results, suggesting that selective learning parameters are differentially modulated by dopaminergic agonists and antagonist treatments. Functional neuroimaging may provide valuable insight into how chronic cocaine use might change the neural networks implicated in associative learning.

### Conclusion

We show that patients with CUD have deficits in the reinforcement learning parameter of learning rate, which were neither related to structural connectivity in the ‘goal-directed’ pathway nor explained their strong habit bias. Moreover, we also identified significantly reduced integrity in white matter structure in brain structures implicated in habit formation, which also did not explain CUD patients’ strong habit bias. Our results are relevant to the hypothesis that drug addiction results in an imbalance between goal-directed and habitual control over behaviour.

## Electronic supplementary material

ESM 1 (PDF 1078 kb)
