# Supplementary material for: Impairments in reinforcement learning do not explain enhanced habit formation in cocaine use disorder
Source: Psychopharmacology (Berl). 2019 Aug 1;236(8):2359–71. doi: 10.1007/s00213-019-05330-z (PMC6695345; doi:10.1007/s00213-019-05330-z)
Supplement: Supplementary file 1 — (PDF 1078 kb) [file 213_2019_5330_MOESM1_ESM.pdf]

Supplemental materials for:

Impairments in reinforcement learning do not explain enhanced habit formation in cocaine use disorder

Lim TV[1], Cardinal RN[1,2,3], Savulich G[1,2], Jones PS[1], Moustafa AA[4], Robbins TW[1,2], Ersche KD[1,2]

Word Count: 2038

Number of Figures: 4

Number of Tables: 4

**Parameters**	**Posterior differences [mean (95% HDI)]**		
	**CUD – HC**	**CUD^+ – HC**	**CUD^+ – CUD**
Learning rate	-0.027 (-0.062, 0.004)	-0.039 (-0.071, -0.012)*	0.012 (-0.013, 0.041)
Reinforcement sensitivity	1.57 (-1.55, 4.88)	0.701 (-1.80, 3.69)	0.868 (-2.52, 4.57)
Perseveration	0.040 (-0.111, 0.193)	-0.058 (-0.185, 0.065)	0.098 (-0.051, 0.248)

CUD: Patients with cocaine without opioid use disorder (n=22)

HC: healthy control volunteers (n=55)

CUD^+: Patients with cocaine + opioid use disorder (n=48)

*probability of non-zero difference, pNZ > 0.95 ($0 \notin$ 95% HDI)

Table S1: Results for reinforcement learning analyses including patients with comorbid opioid use disorder. To ascertain whether opioid use disorder contributed towards reinforcement learning impairments, we fitted the winning reinforcement learning model, but with an extra a priori defined subgroup: patients with cocaine and opioid use disorder. Importantly, results show that there were no group differences within patients on all parameters. Although there is a group difference observed between the groups HC and CUD^+, this difference may largely be driven by the sample size.

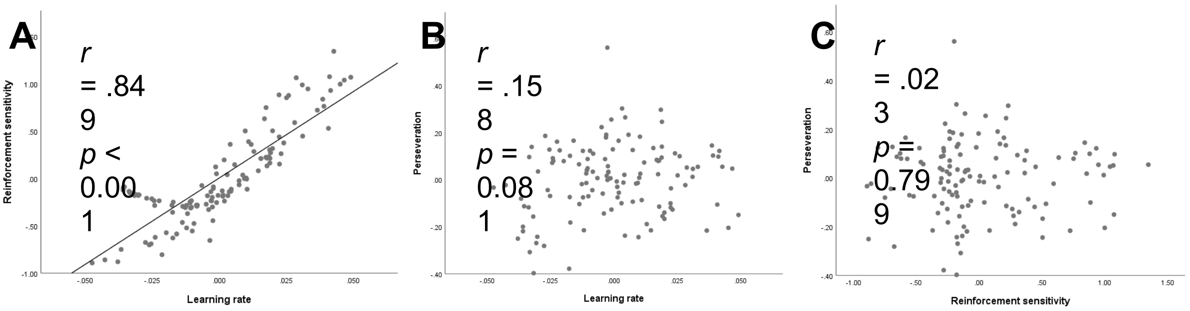

Figure S1: Scatter plots showing the correlations and distributions of the parameters from the winning model across subjects. **A)** Reinforcement sensitivity and learning rate. **B)** Learning rate and perseveration. **C)** Perseveration and reinforcement sensitivity. Each point represents a subject.

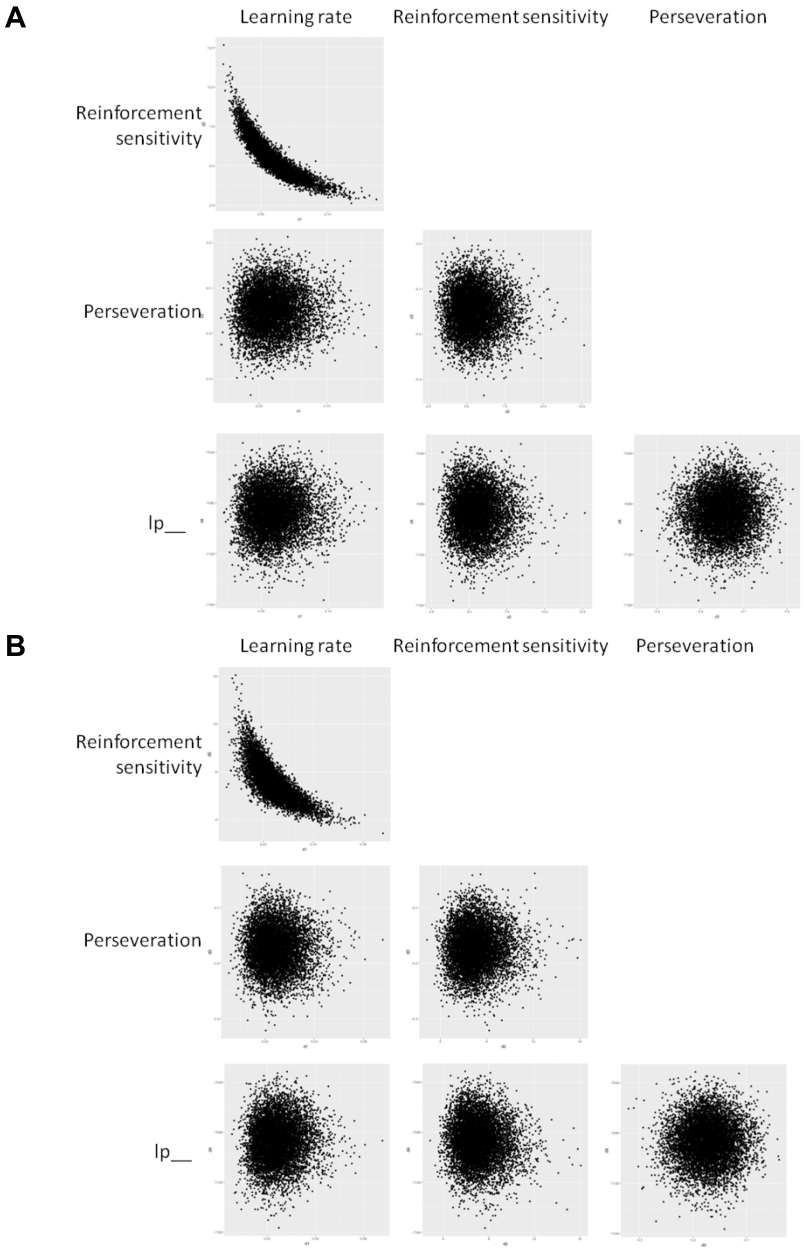

Figure S2: correlations between group-level parameter values from the winning model across iterations. Each point represents one iteration of the winning model. The "lp__" value is Stan's lp__ variable, the log posterior density up to a constant [3]. **(A)** Control subjects. **(B)** CUD subjects.

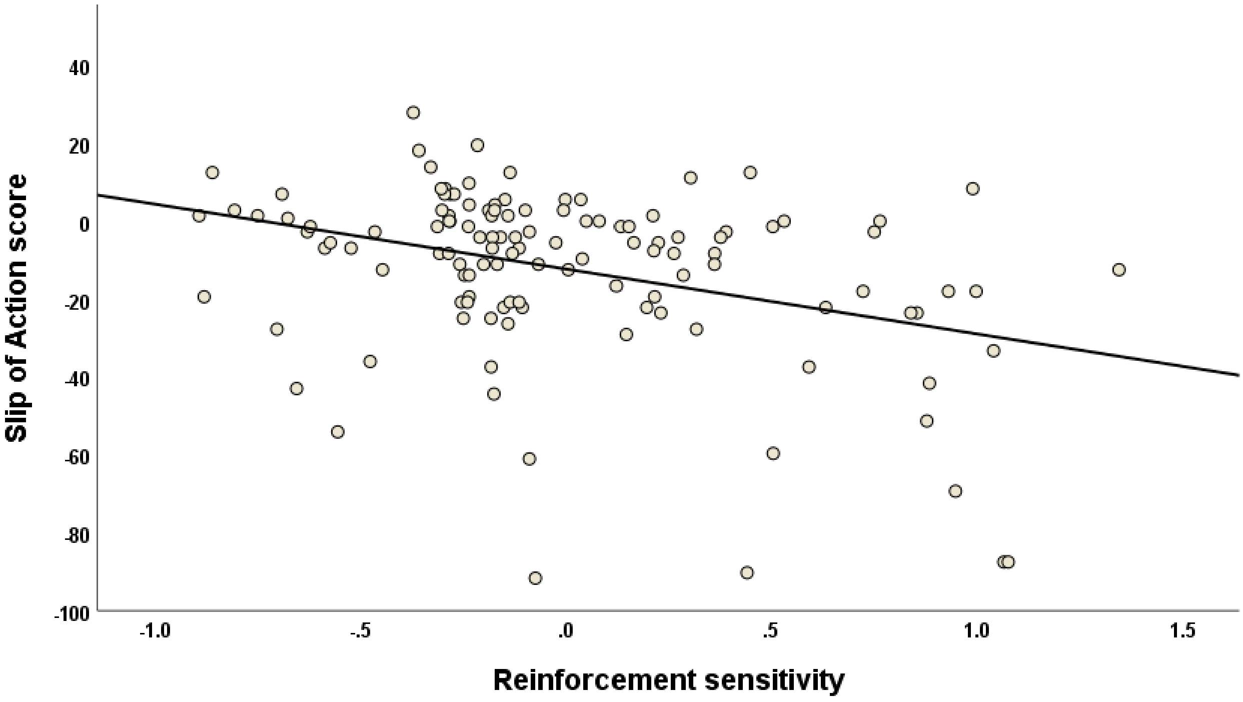

Figure S3: Scatter plot of the relationship between the reinforcement sensitivity parameter (from the winning model) and slip-of-action score (habit bias; behavioural response to outcome devaluation). As reported in the manuscript, reinforcement sensitivity, along with group status, jointly explained 25% of the variance in habitual responding.

Simulation of behavioural data

To determine the validity of the winning RL model, we simulated trial-by-trial data for 200 participants (100 participants per group) from the group means of the posteriors. The simulated data share an identical structure with the original task setup. Each simulated participant data has 96 trials with random assignments of stimulus–correct response mapping. Trial-by-trial responses were generated based on choice probabilities from the RL and softmax equations as reported in the main text of the manuscript. Following the analysis in Ersche et al. (2016), percentage learning accuracy was computed for each participant. Independent-samples t-test confirmed that we replicate the findings – appetitive discrimination learning performance in the CUD group was poorer to that of healthy volunteers ($t_{198} = 5.08$, $p < .001$).

General two-system computational model of goal-directed and habitual responding

We implemented a computational model representing the core features of instrumental learning [1], namely goal-directed action and stimulus–response (S–R) habits (but not including Pavlovian–instrumental transfer), plus response perseveration as before.

We define notation as shown in **Table S2**.

Table S2: notation for the two-system instrumental computational model

Term	***Description***	***Category*** *(LTM long-term memory, WM working memory)*
n_S	Number of stimuli. Integer.	Constant
n_A	Number of actions (including one for "no action", required to predict the consequences of inaction and to choose not to act, if permitted). Integer.	Constant
n_O	Number of outcomes. Integer.	Constant
α^O	Outcome (action–outcome contingency) learning rate for the goal-directed action system. Scalar.	Parameter
α^H	Learning rate for the habit system. Scalar.	Parameter
β^G	Inverse temperature parameter representing the effectiveness of the goal-directed action system at driving behaviour. Scalar.	Parameter
β^H	Inverse temperature parameter representing the effectiveness of the habit system. Scalar.	Parameter
β^P	Inverse temperature parameter representing the effectiveness of the response perseveration system. Scalar.	Parameter
t	Current trial number. Integer.	Time
$\boldsymbol{G}$	Current goal-directed action–outcome (A–O) contingencies. An $n_S \times n_A \times n_O$ matrix, mapping discriminative stimuli to A–O contingencies. Starting values are 0.	Subject LTM
$\boldsymbol{v}$	Current instrumental outcome values. Vector of size n_O. The value of outcome o is denoted $\boldsymbol{v}_o$. Starting values are 0.	Subject LTM
$\boldsymbol{H}$	Current stimulus–response (S–R, stimulus–action) habit strengths. An $n_S \times n_A$ matrix mapping stimuli to "action values" (Q values). Starting values are 0.	Subject LTM
$\boldsymbol{s}$	Stimuli: vector of length n_S representing stimulus presence (0) or absence (1) for all stimuli on trial t.	World → subject
$\boldsymbol{C}$	Action–outcome contingencies predicted on trial t by the stimuli currently present, from the tensor dot product of $\boldsymbol{s}$ and $\boldsymbol{G}$; these contingencies can exceed the conventional contingency range [–1, +1]. An $n_A \times n_O$ matrix.	Subject WM
$\boldsymbol{q}^G$	Action "values" (expected value of the action; Q values) for trial t: goal-directed component. Vector of size n_A.	Subject WM
$\boldsymbol{q}^H$	Action "values" for trial t: habit component. Vector of size n_A.	Subject WM
$\boldsymbol{q}^P$	Action "values" for trial t: perseverative component. Vector of size n_A. Defined to contain zeros for all actions, except 1 for the action chosen on the preceding trial (if there was a preceding trial).	Subject WM
$\boldsymbol{a}$	Action tendencies for trial t. Vector of size n_A.	Subject WM
$\boldsymbol{p}$	Probability of making each action on trial t. Vector of size n_A.	Subject WM
a	The selected action (as an index). Integer.	Subject → world
$\boldsymbol{o}$	Representation of which outcomes were obtained on trial t. Binary vector of length n_O containing 1 for outcomes that were obtained and 0 for those that were not.	World → subject
r	Reinforcement value of the outcome(s) obtained. Scalar.	Subject WM
d^H	Reinforcement prediction error (d for discrepancy) for the habit system on trial t. Scalar.	Subject WM
$\boldsymbol{d}^O$	Outcome prediction error for the goal-directed system on trial t. Vector of size n_A.	Subject WM

Actions were determined as follows. Discriminative stimuli (SDs) present ($\boldsymbol{s}$) were combined with previous knowledge of SD-dependent contingencies ($\boldsymbol{G}$) to predict the action–outcome contingencies currently operative ($\boldsymbol{C}$). Combining these contingencies with the value of the outcomes ($\boldsymbol{v}$) gives the declarative expected value of each goal-directed action ($\boldsymbol{q}^G$). Simultaneously, the same environmental stimuli ($\boldsymbol{s}$) act via S–R associations ($\boldsymbol{H}$) to drive actions habitually ($\boldsymbol{q}^H$); this is a procedural rather than a declarative representation [1] but the quantity $\boldsymbol{q}^H$ reflects the "expected value" of actions based on past experience (in a different sense to a declarative expectation). Perseveration produces a further direct drive ($\boldsymbol{q}^P$) towards the most recently selected action.

$$\boldsymbol{C}=\boldsymbol{s}\cdot\boldsymbol{G}$$
$$\boldsymbol{q}^G=\boldsymbol{C}\cdot\boldsymbol{v}$$
$$\boldsymbol{q}^H=\boldsymbol{s}\cdot\boldsymbol{H}$$
$$\boldsymbol{a}=\beta^G\boldsymbol{q}^G+\beta^H\boldsymbol{q}^H+\beta^P\boldsymbol{q}^P$$
$$\boldsymbol{p}=\text{softmax}(\boldsymbol{a})=\frac{e^{\boldsymbol{a}}}{\sum e^{\boldsymbol{a}}}$$

For constrained choices, such as two-choice trials not permitting a "non-response" action, the softmax was calculated across valid responses only (with $\boldsymbol{p}_a = 0$ for all actions not permitted).

The goal-directed system learned as follows. Instrumental contingency learning was driven by calculating an outcome prediction error $\boldsymbol{d}^O$ for all outcomes, as the difference between obtained outcomes ($\boldsymbol{o}$) and predicted outcomes (action–outcome contingencies for the chosen action: $\boldsymbol{C}_{a,*}$). A–O contingencies predicted for the chosen action by stimuli currently present ($\boldsymbol{G}_{\boldsymbol{s},a}$) were then updated using this prediction error:

$$\boldsymbol{d}^O=\boldsymbol{o}-\boldsymbol{C}_{a,*}$$
$$\Delta\boldsymbol{G}_{\boldsymbol{s},a}=\alpha^O\boldsymbol{d}^O$$

A more general form might include instrumental incentive learning [1] in which values for obtained outcomes are changed ($\Delta\boldsymbol{v}_{\boldsymbol{o}}$) according to an outcome value error (d^V, the obtained reinforcement r minus the total value predicted for the obtained outcomes, $\boldsymbol{o}\cdot\boldsymbol{v}$) and a learning rate α^V:

$$d^V=r-\boldsymbol{o}\cdot\boldsymbol{v}$$
$$\Delta\boldsymbol{v}_{\boldsymbol{o}}=\alpha^V d^V$$

In the situation of a single outcome and $\alpha^V = 1$, this reduces to direct assignment of the reinforcement value to the obtained outcome. However, in the present task and model, the situation was even simpler: outcomes values were directly instructed, and so α^V was not considered.

Habit learning was as follows. S–R associations between stimuli present ($\boldsymbol{s}$) and the action performed (a) were updated according to the reinforcement prediction error d^H, the difference between the reinforcement obtained (r) and the reinforcement predicted by the chosen action ($\boldsymbol{q}^H_a$):

$$d^H=r-\boldsymbol{q}_a^H$$
$$\Delta\boldsymbol{H}_{\boldsymbol{s},a}=\alpha^H d^H$$

Specific implementation for the slips-of-action task

We modelled data from both relevant phases of the original task [2] (phase A, appetitive learning, and phase C, slip-of-action responding following outcome devaluation). We did not model the outcome–action contingency assessment (phase B), which did not not involve reinforcement feedback. We did not analyse the control task in phase D (responding to discriminative stimuli that themselves were or were not "devalued").

Reinforcing outcomes were given a notional and arbitrary value of +5 points. As described above, outcome devaluation was represented by direct instantaneous instruction in the model, reflecting the direct instruction in the slips-of-action task ("these animals are sick, avoid them"); these outcomes were temporarily devalued to –5 points. As feedback was not provided in this phase of the task and the behavioural task was framed to avoid learning, the goal-directed system was prevented from learning during these trials. The task was framed as a go/no-task and choosing the "originally correct" side was scored as a "response" (as per Figure 1C[right] of Ersche et al. 2016 [2]). Choosing the other side, or not acting at all, was scored as a "non-response". A goal-directed subject will respond less when the relevant outcome is devalued; a habit-based subject will not alter its behaviour.

We constrained the general computational model further, via the restriction $\alpha^G = \alpha^H$, as the behavioural task did not permit differential assessment of the learning rates of instrumental and habitual systems (such that separate alpha values would lead to overfitting and did so in pilot modelling); different contributions of the two systems are therefore reflected primarily in β^G and β^H.

The behavioural task, involving explicit instructions to humans, is ambiguous as to whether it would lead to ongoing habit learning during the test phase (arguments might include: responding is to the same manipulanda, so ongoing habit learning might be expected; or, the instruction change sets up a different context, sharply altering the stimuli participating in S–R learning). Consequently, we tested two versions of the task: in one, habit learning was assumed to continue during the slips-of-action phase ("habit learning at test", HLAT); in another, it was assumed that no further habit learning occurred ("no habit learning at test", NHLAT).

Bayesian hierarchy and simulations

The group-level structure of the Bayesian model was as before, with a per-group mean and a common intersubject standard deviation for each parameter.

Priors were as shown in **Table S3**.

Table S3: priors for the two-system instrumental computational model.

Parameter	Prior for parameter	Prior for intersubject standard deviation
α	Beta(1.2, 1.2)	Half-normal(0, 0.17)
β^G, β^H	Gamma(shape = alpha = 4.82, rate = beta = 0.88)	Half-normal(0, 2)
β^P	Normal(0, 1)	Half-normal(0, 2)

We used a variational Bayes approximation to obtain posterior parameter distributions, via Stan's ADVI [automatic differentiation variational inference] algorithm [3, 4].

Results

Results are shown in **Table S4**. The behavioural task [2] was ill-specified as to whether further S–R learning would occur in the outcome devaluation test phase, and this had a potentially important impact on the assessment of learning rates in the two-system model: if learning was assumed to occur, the model suggested faster learning in the CUD group, and if not, it suggested slower learning. Interpretation of learning rate from this model is therefore more complex (see main text for discussion) but the results reflect slower learning in the first phase and a likely confound between the effects of outcome devaluation and those of extinction in measuring the effect of learning in the second phase. The models were consistent, however, in showing a reduced impact of the goal-directed action system (lower β^G) in the CUD group; no difference in the impact of the habitual system (no difference in β^H); and a greater tendency to perseverate (β^P) (or, strictly, a lesser tendency to avoid a recently chosen option, since β^P estimates were negative). Interparameter scatterplots are shown in **Figure S3.**

Table S4: results for the two-system instrumental computational model.

Condition	Parameter	Control group (posterior mean and 95% HDI)	CUD group (posterior mean and 95% HDI)	CUD – control (posterior mean and 95% HDI); **bold** indicates an HDI excluding zero (posterior probability >95% of a difference between groups)
No S–R learning during assessment phase ("no habit learning at test"; NHLAT)				
	α	0.100 [0.090, 0.111]	0.041 [0.037, 0.045]	**–0.059 [–0.070, –0.049]**
	β^G	0.323 [0.297, 0.351]	0.280 [0.247, 0.316]	**–0.043 [–0.083, –0.001]**
	β^H	0.004 [0.002, 0.007]	0.008 [0.004, 0.013]	0.004 [–0.001, 0.010]
	β^P	–0.215 [–0.251, –0.172]	–0.024 [–0.059, 0.011]	**0.190 [0.138, 0.240]**
S–R learning allowed during assessment phase ("habit learning at test"; HLAT)				
	α	0.035 [0.031, 0.038]	0.096 [0.085, 0.109]	**0.062 [0.050, 0.074]**
	β^G	0.653 [0.616, 0.693]	0.182 [0.166, 0.199]	**–0.471 [–0.514, –0.433]**
	β^H	0.002 [0.001, 0.004]	0.001 [0.000, 0.003]	–0.000 [–0.002, 0.002]
	β^P	–0.255 [–0.293, –0.215]	–0.096 [–0.134, –0.057]	**0.159 [0.105, 0.213]**

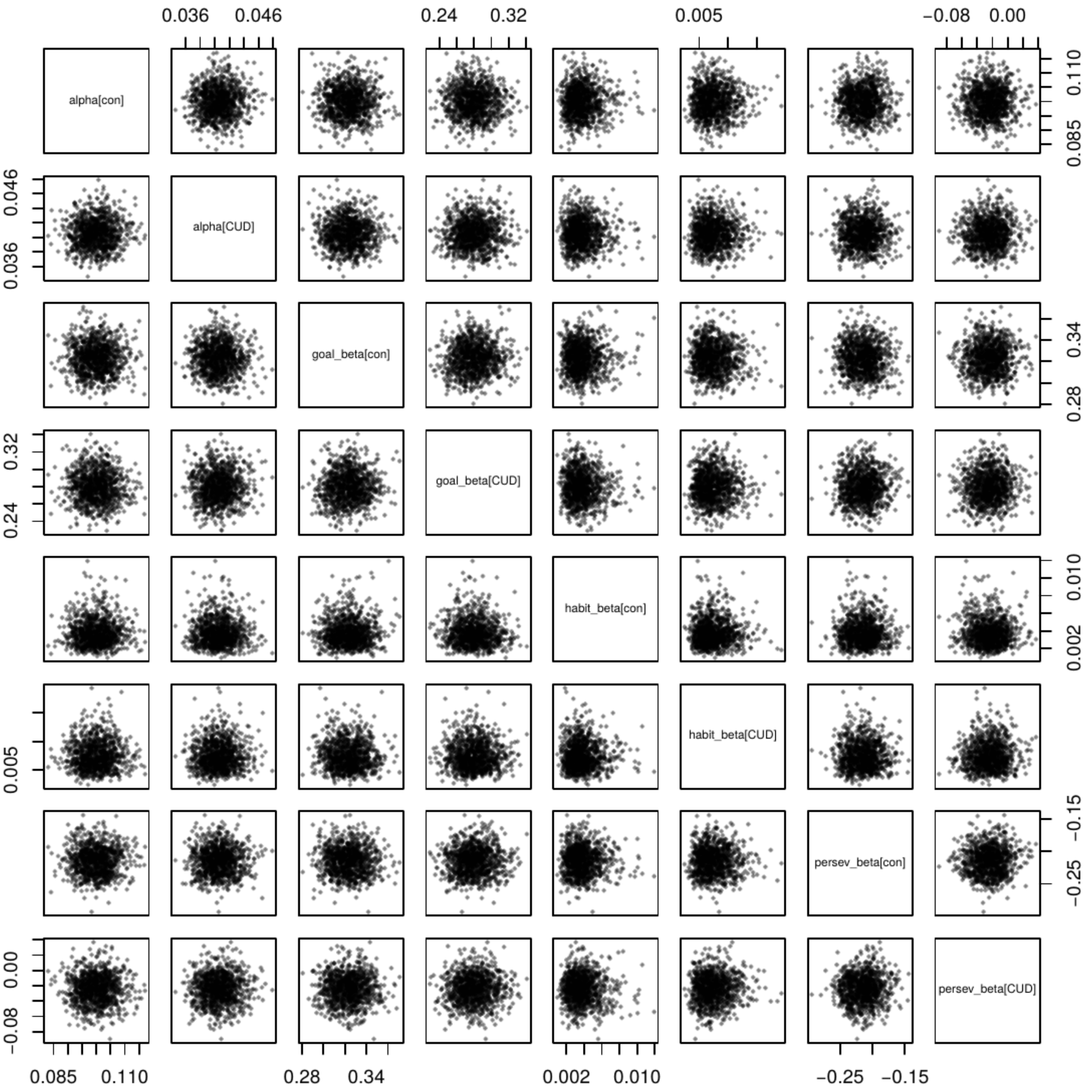

Figure S3: correlations between group-level parameter estimates from the two-system instrumental NHLAT model. Each point represents an iteration of the simulation.

References

[1] Cardinal RN, Parkinson JA, Hall J, Everitt BJ. Emotion and motivation: the role of the amygdala, ventral striatum, and prefrontal cortex. *Neuroscience and Biobehavioral Reviews* 26: 321–52.

[2] Ersche KD et al. (2016). Carrots and sticks fail to change behavior in cocaine addiction. *Science* 352: 1468–71.

[3] Stan Development Team (2019). *Stan Reference Manual (Version 2.19)*, https://mc-stan.org/.

[4] Kucukelbir A, Ranganath R, Gelman A, Blei DM (2015). Automatic variational inference in Stan. *ArXiv:1506.03431*.
